# Perilipin 5 alleviates HCV NS5A-induced lipotoxic injuries in liver

**DOI:** 10.1186/s12944-019-1022-7

**Published:** 2019-04-06

**Authors:** Jin Zhang, Xing Gao, Yuan Yuan, Chao Sun, Yuanlin Zhao, Liming Xiao, Ying Yang, Yu Gu, Risheng Yang, Peizhen Hu, Lijun Zhang, Chao Wang, Jing Ye

**Affiliations:** 1 State Key Laboratory of Cancer Biology, Department of Pathology, Xijing Hospital and School of Basic Medicine, Fourth Military Medical University, No.169, Changle West Road, Xi’an, Shaanxi 710032 People’s Republic of China; 2 Department of Neurology, Tangdu Hospital, the Fourth Military Medical University, Xi’an, Shaanxi 710032 People’s Republic of China; 3 Department of Clinical Laboratory Medicine, Tangdu Hospital, the Fourth Military Medical University, Xi’an, Shaanxi 710038 People’s Republic of China; 4 Department of Pathology, The General Hospital of Western Theater Command, No. 270, Tianhui Road, Rongdu Avenue, Chengdu, 610083 People’s Republic of China

**Keywords:** Lipid droplet, Perilipin 5, Hepatitis C virus, Nonstructural protein 5A, Lipolysis

## Abstract

**Background:**

The homeostasis of lipid droplets (LDs) plays a crucial role in maintaining the physical metabolic processes in cells, and is regulated by many LD-associated proteins, including perilipin 5 (Plin5) in liver. As the putative sites of hepatitis C virus (HCV) virion assembly, LDs are vital to viral infection. In addition, the hepatic LD metabolism can be disturbed by non-structural HCV proteins, such as NS5A, but the details are still inexplicit.

**Methods:**

HCV NS5A was overexpressed in the livers and hepatocytes of wild-type and *Plin5*-null mice. BODIPY 493/503 and oil red O staining were used to detect the lipid content in mouse livers and hepatocytes. The levels of lipids, lipid peroxidation and inflammation biomarkers were further determined. Immunofluorescence assay and co-immunoprecipitation assay were performed to investigate the relationship of Plin5 and NS5A.

**Results:**

One week after adenovirus injection, livers expressing NS5A showed more inflammatory cell aggregation and more severe hepatic injuries in *Plin5*-null mice than in control mice, which was consistent with the increased serum levels of IL-2 and TNF-α (*P* < 0.05) observed in *Plin5*-null mice. Moreover, Plin5 deficiency in the liver and hepatocytes aggravated the elevation of MDA and 4-HNE levels induced by NS5A expression (*P* < 0.01). The triglyceride (TG) content was increased approximately 25% by NS5A expression in the wild-type liver and hepatocytes but was unchanged in the *Plin5*-null liver and hepatocytes. More importantly, Plin5 deficiency in the liver and hepatocytes exacerbated the elevation of non-esterified fatty acids (NEFAs) stimulated by NS5A expression (*P* < 0.05 and 0.01 respectively). Using triacsin C to block acyl-CoA biosynthesis, we found that Plin5 deficiency aggravated the NS5A-induced lipolysis of TG. In contrast, Plin5 overexpression in HepG2 cells ameliorated the NS5A-induced lipolysis and lipotoxic injuries. Immunofluorescent staining demonstrated that NS5A expression stimulated the targeting of Plin5 to the surface of the LDs in hepatocytes without altering the protein levels of Plin5. By co-IP, we found that the N-terminal domain (aa 32–128) of Plin5 was pivotal for its binding with NS5A.

**Conclusions:**

Our data highlight a protective role of Plin5 against hepatic lipotoxic injuries induced by HCV NS5A, which is helpful for understanding the steatosis and injuries in liver during HCV infection.

## Background

Hepatitis C virus (HCV), which infects approximately 2.5% of the world’s population [1], causes a spectrum of diseases ranging from liver steatosis, fibrosis, cirrhosis, and hepatocellular carcinoma (HCC) as an end-liver stage [2]. In liver cells, HCV hijacks host lipid metabolism and modulates the membrane dynamics to facilitate its own replication and assembly for its propagation [3]. The most important feature of chronic HCV infection is disordered lipid metabolism, as estimated, for approximately 50–60% chronically infected individuals diagnosed with liver steatosis [4].

HCV RNA is replicated in special membranous structures, and the replication complex is then recruited into adjacent lipid droplets (LDs) in a core-dependent way [5], consequently, the intracellular lipid levels and fatty acid composition have a strong influence on HCV life cycle. Although all non-structural proteins are involved in HCV replication, NS5A is absolutely a central determinant in the replication complex [6, 7], and it has also been reported to augment the transcriptional activity and gene expression of PPAR-γ to stimulate lipid accumulation [8]. Recently, it was reported that NS5A could interact with tail-interacting protein 47 (TIP47, Plin3), probably for the purpose of increasing its affinity with LDs [9–11]. As a member of the perilipin family, TIP47 promotes the maturation of LDs and regulates the incorporation of triglycerides (TGs) into LDs [12].

Regulated by many LD-associated proteins, LDs occupy a central role in maintaining lipid metabolic homeostasis. Among them, perilipin 5 (Plin5) is a vital member of the perilipin family expressed specifically in oxidative tissues such as the heart, brown adipose tissue (BAT), skeletal muscle and liver [13]. *Plin5*-deficient mice showed the decreased accumulation of the LDs, and the results in vitro also suggested that Plin5 is responsive to high lipid oxidative metabolism to protect LD storage against neutral lipases and maintain stability by reducing lipotoxic liver injury [14, 15]. However, the role of Plin5 in the HCV-infected liver is still inexplicit. Hence, *Plin5*-deficient mice were used to investigate the role of Plin5 in NS5A-induced lipid dysmetabolism and lipotoxic injuries in the liver.

## Methods

### Adenovirus and animal studies

Adenovirus expressing HCV NS5A (NC_004102.1, GenBank) were generated using AdEasy-1 system (Clontech) as previously described [16]. *Plin5* knockout mice were established using a standard gene targeting procedure based on a homologous recombination strategy as previously described [15]. Male *Plin5*-null mice and control wild-type mice that were 8-week-old were administered 1 × 10^8^ plaque forming units (pfu) of adenovirus expressing NS5A or a blank control in saline through their tail veins. One week after infection, the mice were euthanized, and the livers were excised.

### Plasmid construction

DNA encoding full-length and truncated Plin5 (NM_025874.3) was generated by PCR using specific primers. These DNA fragments were subcloned into the pCMV5-FLAG or pCMV5-HA vectors to generate the FLAG- or HA-tagged full-length Plin5-expressing plasmids, and GFP-tagged truncated Plin5 was generated by subcloning the fragments into the pEGFP-N2 vector (Clontech). The truncated Plin5 constructs included the following amino acids: 1–128, 1–212, 1–260, 1–382, 1–400 and △32–128.

### Cell culture and transfection

HEK 293 T cells were maintained in Dulbecco’s modified Eagle’s medium (DMEM, Gibco, USA) containing 10% foetal bovine serum (FBS, ExCell Bio, China) and 100 units/ml penicillin/streptomycin. Cells were cultivated in humidified air in 5% CO_2_ at 37 °C. Polyethylenimine (PEI, Sigma) transfection was performed, and the cells were harvested 24 h after transfection.

### Primary hepatocyte isolation and culture

The mice were anaesthetized with 0.4% pentobarbital sodium, the abdominal inferior vena cava (IVC) was exposed, and a catheter was placed into the IVC. After cut the portal vein, the liver was perfused with perfusion medium (120 mM NaCl, 24 mM NaHCO_3_, 20 mM glucose, 5 mM HEPES, 480 μM KCl, 120 μM MgSO_4_, 120 μM KH_2_PO_4_, 0.1 mM EGTA, pH 7.4) for 15 min at 3 ml/min. Then, the liver was digested with digest medium (120 mM NaCl, 24 mM NaHCO_3_, 20 mM glucose, 5 mM HEPES, 480 μM KCl, 120 μM MgSO_4_, 120 μM KH_2_PO_4_, 1.4 mM CaCl_2_, 0.05% collagenase, pH 7.4). After perfusion, the liver was excised, and the hepatocytes were dispersed and filtered using a 70 μm cell strainer (Millipore, USA). The cells were washed with 50 ml of ice-cold serum-free DMEM, followed by centrifugation at 50×*g* for 5 min at 4 °C. The procedures above were repeated again, DMEM (Gibco, USA) containing 10% foetal bovine serum (Gibco, USA) was then added, and the primary hepatocytes were incubated in 5% CO_2_ at 37 °C.

### Biochemical parameters

The peripheral blood was collected before the mice were sacrificed, and the serum levels of alanine transaminase (ALT) and aspartate transaminase (AST) were quantified using an automatic biochemical analyser. The levels of IL-2 and tumour necrosis factor (TNF-α) in the serum were detected by using enzyme-linked immunosorbent assay (ELISA) kits (69–99852, 69–99985, MSK, China) according to the manufacturer’s instructions.

### Immunohistochemistry and immunofluorescence

Formalin-fixed, paraffin-embedded liver tissues were cut into 3-μm-thick sections. Hydrated sections were boiled for 15 min in citric acid buffer (pH 6.0). After blocking endogenous peroxidase and non-specific antigens, the sections were incubated with anti-F4/80 (70076 T, Cell Signaling Technology) or anti-CD3 (ab5690, Abcam) antibodies. The tissue sections were then incubated with relevant secondary antibody (Maixin Biotechnologies, China). Finally, diaminobenzidine (DAB, Maixin Biotechnologies) was added as a substrate.

For immunofluorescent staining, mouse primary hepatocytes were seeded on glass coverslips 72 h after infection with adenovirus. After washing with PBS, the cells were directly fixed in 4% paraformaldehyde for 20 min, followed by permeabilization with 0.5% Triton X-100 for 15 min. Then, the cells were stained with diluted rabbit anti-HA monoclonal antibody (ABclonal, USA) and mouse anti-Plin5 antibody (homemade) [17] overnight at 4 °C, followed by donkey anti-rabbit IgG (Alexa Fluor 594 conjugate, Invitrogen) and donkey anti-mouse IgG (Alexa Fluor 488 conjugate, Invitrogen). The nuclei were counterstained with Hoechst 33258 (Invitrogen, USA). The fluorescence was viewed and imaged under a fluorescence microscope (Olympus IX71).

### Oil red O staining

The frozen liver sections (10 μm) were fixed with 10% formalin in PBS for 30 min at room temperature. The sections were subsequently washed with 60% isopropanol for several times and then treated with 0.5% freshly prepared oil red O solution for 15 min. Then the sections were rinsed several times with 60% isopropanol and imaged using a light microscope (Olympus BX53).

### BODIPY 493/503 staining

For this assay, primary hepatocytes were seeded onto glass coverslips in 12-well plates (approximately 1 × 10^5^ cells/well). After 12 h, the cells were infected with adenovirus (MOI = 50) expressing NS5A (Ad-NS5A) or blank control (Ad-Null). After adherence, the cells were incubated with 200 μΜ OA (oleic acids, O3008, Sigma). The cells were incubated for another 24 h and then fixed with 4% formaldehyde at room temperature. After washing with PBS, the cells were incubated with BODIPY 493/503 at a working concentration of 5 μg/ml for 20 min at room temperature. After incubation, the cells were washed twice with PBS and counterstained with Hoechst 33258 (Invitrogen, USA). The slides were mounted and then imaged using a fluorescence microscope (Olympus IX71).

### Lipid peroxidation assay

Lipid peroxidation levels in liver homogenates were determined by measuring the levels of malondialdehyde (MDA) and 4-hydroxynonenal (4-HNE) adducts, two principal parameters of lipid oxidative stress. MDA was measured using an assay kit (A003–4,Jiancheng Bioengineering) following the manufacturer’s instructions. The 4-HNE adducts were analysed by the Oxiselect HNE-His Adduct ELISA kit (Cell Biolabs, USA).

### Lipid droplet isolation

Lipid droplets were isolated as previously reported [18]. Briefly, the infected livers and primary hepatocytes were homogenized in ice-cold HLM buffer (20 mM Tris-HCl, 1 mM EDTA, 10 mM sodium fluoride, 1 mM PMSF, pH 7.4) with a Dounce tissue grinder (Kimble Chase, USA). The homogenate was centrifuged at 1000⨯*g* to remove the nuclei, and the post-nuclear supernatant was adjusted with ice-cold HLM buffer containing 60% sucrose (20% final sucrose concentration), HLM buffer with 5% sucrose and sucrose-free HLM buffer successively in a bottom-up approach. After centrifugation for 30 min at 28,000×*g* and 4 °C, the floating opaque lipid layer was collected through a discontinuous sucrose gradient. After delipidating with acetone and ether, the LD proteins were dissolved in SDS-PAGE loading buffer, and the samples were detected by immunoblotting.

### Quantitation of intracellular triglyceride (TG) and non-esterified fatty acids (NEFAs)

A total of 100 mg liver tissues and 1 × 10^7^ primary hepatocytes were homogenized or suspended in 2 ml PBS, and the total lipids were extracted with 6 ml hexane/isopropanol mixture (*v*/v = 3:2). After centrifugation, the upper phase was collected into a glass tube and dried under nitrogen steam at 60 °C. The extracted lipids were dissolved in 200 μl of 2% Triton X-100 with vigorous vortexing. The triglyceride contents were determined by a kit (AM544, Wako), and the non-esterified fatty acid (NEFA) contents were measured by a kit (MAK044-1kt, Sigma). The protein pellets were dissolved in 2 M KOH solution, and the protein concentrations were measured by the BCA method (Beyotime Biotechnology, China).

### Co-immunoprecipitation (co-IP) assay

HEK 293 T cells were transfected with plasmids expressing full-length or truncated Plin5 and NS5A, and the hepatocytes were infected with the indicated adenovirus respectively. The cells in 60 mm dishes were washed with ice-cold PBS and scraped into 1 ml of co-IP lysis buffer (20 mM Tris-HCl pH 7.4, 150 mM NaCl, 2.5 mM sodium pyrophosphate, 1.0 mM β-glycerophosphate, 1.0% Triton X-100, 1.0 mM EDTA, 1.0 mM EGTA, 1.0 mM PMSF) on ice. After ultrasonification, the lysates were centrifuged at 12,000 rpm for 30 min at 4 °C. Ten microliters of washed protein A/G agarose beads (sc-2003, Santa Cruz) with 50 ng anti-FLAG (KM8002, SunGene), anti-HA (AE008, ABclonal) or anti-Plin5 (homemade) antibodies were added as indicated to the supernatant and incubated overnight at 4 °C. After incubation, the beads were pelleted by centrifuging at 3000 rpm for 2 min at 4 °C. After washing with ice-cold co-IP lysis buffer, the agarose beads were resuspended in 50 μl SDS-PAGE loading buffer. The sample (15 μl per lane) was analysed by SDS-PAGE and the relevant proteins were detected by immunoblotting.

### Reactive oxygen species (ROS) determination

Primary hepatocytes and HepG2 cells were seeded on 6-well plates, and then infected with control adenoviruses (Ad-Null) or NS5A adenoviruses (Ad-NS5A) respectively. After incubation overnight, 200 mM OA was added for another 24 h to promote the lipid accumulation. The hepatocytes or HepG2 cells were incubated with carboxy-2′,7′-dichloro-dihydro-fluorescein diacetate (DCFH-DA, Beyotime) for 20 min at 37 °C. The medium was discarded, the cells were washed three times with serum-free DMEM, and the fluorescence was measured under a fluorescence microscope (Olympus IX71).

### Lipolysis determination

Determining the rate of triacylglycerol hydrolysis is an established procedure to estimate the lipolysis [19]. 200 μM OA was supplied to the primary hepatocytes infected with Ad-NS5A or Ad-Null to increase TG accumulation for 24 h. Then the supplemental fatty acids were withdrawn, and the hepatocytes were treated with 5 μM triacsin C (sc-200574A, Santa Cruz) to inhibit further TG synthesis. The hepatocytes were harvested at various times, and the relative hydrolysis rate were indicated by the percentage of remaining triacylglycerol content divided by the triacylglycerol content immediately following the incubation with triacsin C.

### Immunoblotting

The protein samples were quantified using a BCA protein assay kit (Beyotime Biotechnology, China). Proteins were subjected to SDS-PAGE and electroblotted onto PVDF membranes (Millipore, USA). The membranes were blocked with 5% non-fat milk before incubation with the anti-FLAG (KM8002, SunGene Biotech), anti-HA (AE036, ABclonal), anti-GFP (AE011, ABclonal), anti-Plin5 (homemade) [17], anti-ATGL (2138S, CST) and anti-β-tubulin (KM9003, SunGene Biotech) antibodies followed by incubation with horseradish peroxidase-conjugated secondary antibodies (115–035-003, 111–035-003, Jackson) or HRP-Protein A (#7074, CST). Blots were visualized using an enhanced chemiluminescence substrate and exposure to X-ray film.

### Statistical analysis

All data are presented as the mean ± SEM. The differences between groups were analysed by unpaired Student’s *t*-test using SPSS 22.0 software (SPSS, USA). Statistical significance was indicated as *P* < 0.05.

## Results

### Plin5 deficiency aggravated HCV NS5A-induced hepatic injuries

**Fig. 1:**
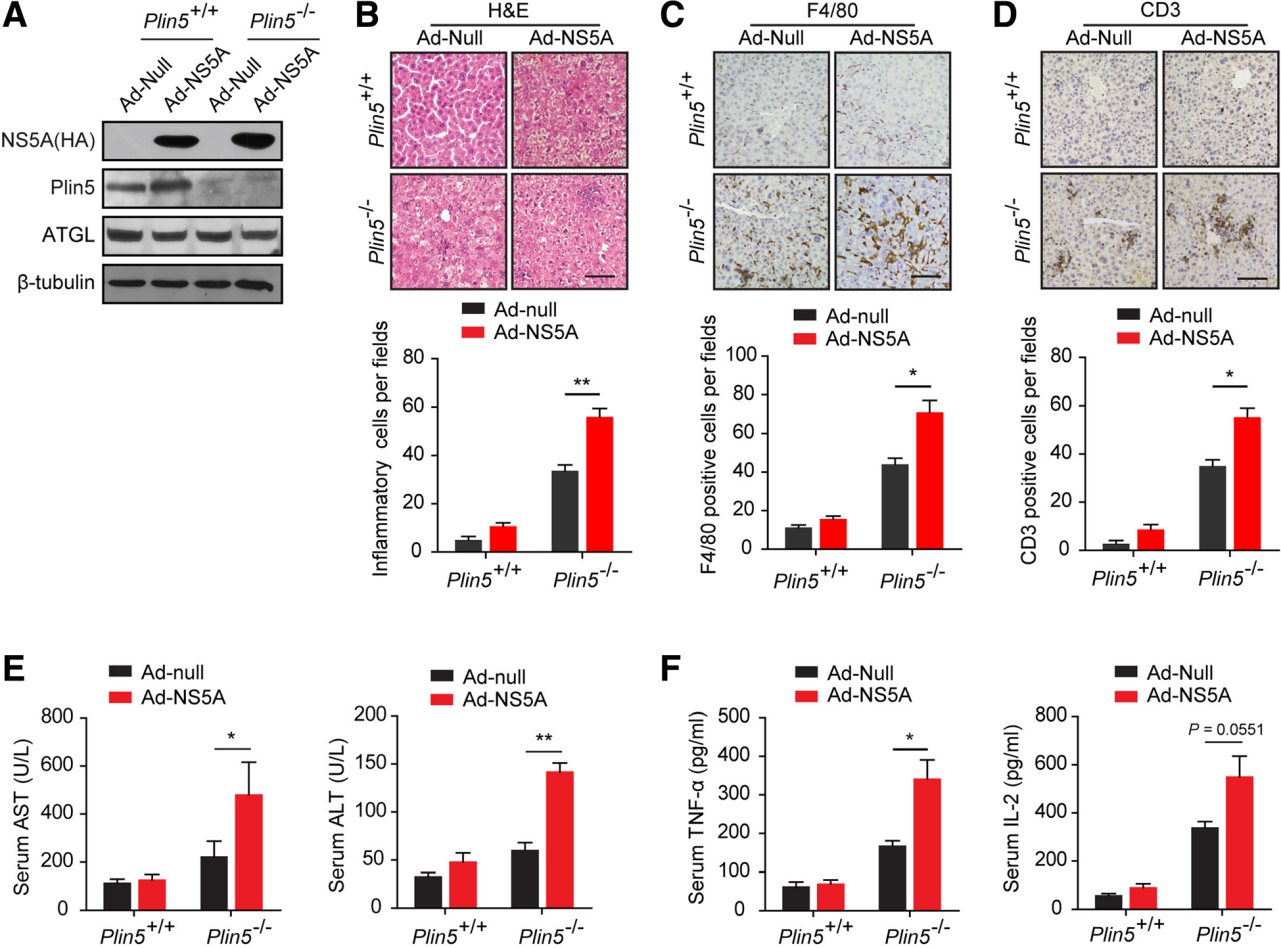
Plin5 deficiency aggravated HCV NS5A-induced hepatic injuries. Adenovirus containing NS5A (Ad-NS5A) or the control adenovirus (Ad-Null) were administrated to the wild-type and *Plin5*-null mice through tail veins respectively (*n* = 4). After 1 week, their livers were removed and the bloods were collected. **a** The expression of NS5A, Plin5 and ATGL in the livers was analysed by immunoblotting. **b** H&E staining showed that NS5A induced more severe hepatic injuries and more inflammatory cell infiltration in the *Plin5*-null livers than in wild-type livers. Scale bar = 50 μm. c & d Immunohistochemical staining indicated that NS5A significantly increased the infiltration of F4/80-positive macrophages (**c**) and CD3-positive T lymphocytes (**d**) in *Plin5*-null livers compared to that observed in wild-type livers. Scale bar = 50 μm. **e** & **f** The levels of IL-2 (**e**) and TNFα (**f**) in mouse sera were analysed by ELISA kits. *P < 0.05, **P < 0.01. Data are means ± SEs for triplicate samples for each representative experiment

To investigate the role of Plin5 in HCV NS5A-induced lipid dysmetabolism, we administered 8-week-old male wild-type and *Plin5*-null mice adenovirus expressing NS5A by tail vein injection, and Ad-Null served as negative control. One week after injection, immunoblotting verified that NS5A was expressed in both wild-type and *Plin5*-null livers, and the expression of adipose triglyceride lipase (ATGL) remained unchanged (Fig. 1a). H&E staining showed that livers expressing NS5A showed more inflammatory cell aggregation and more severe hepatic injuries in *Plin5*-null mice than the livers in control mice (Fig. 1b). We further investigated the degree of inflammatory responses by immunohistochemistry (IHC) with anti-F4/80 and anti-CD3 antibodies, and the numbers of F4/80-positive macrophages and CD3-positive T lymphocytes were significantly higher in *Plin5*-null livers than in wild-type livers (Fig. 1c &d). Blood analysis showed that NS5A significantly increased the serum alanine transaminase (ALT) and aspartate transaminase (AST) levels in *Plin5*-null mice compared to the levels in control mice, although the ALT levels were slightly elevated in wild-type mice (Fig. 1e). Consistent with the results of F4/80 and CD3 staining, NS5A expression increased the serum levels of IL-2 and TNF-α in *Plin5*-null mice compared to those in control mice (Fig. 1f). Thus, these results revealed that Plin5 deficiency aggravated NS5A-induced hepatic injuries.

### Plin5 alleviated NS5A-induced lipid peroxidation in liver cells

**Fig. 2:**
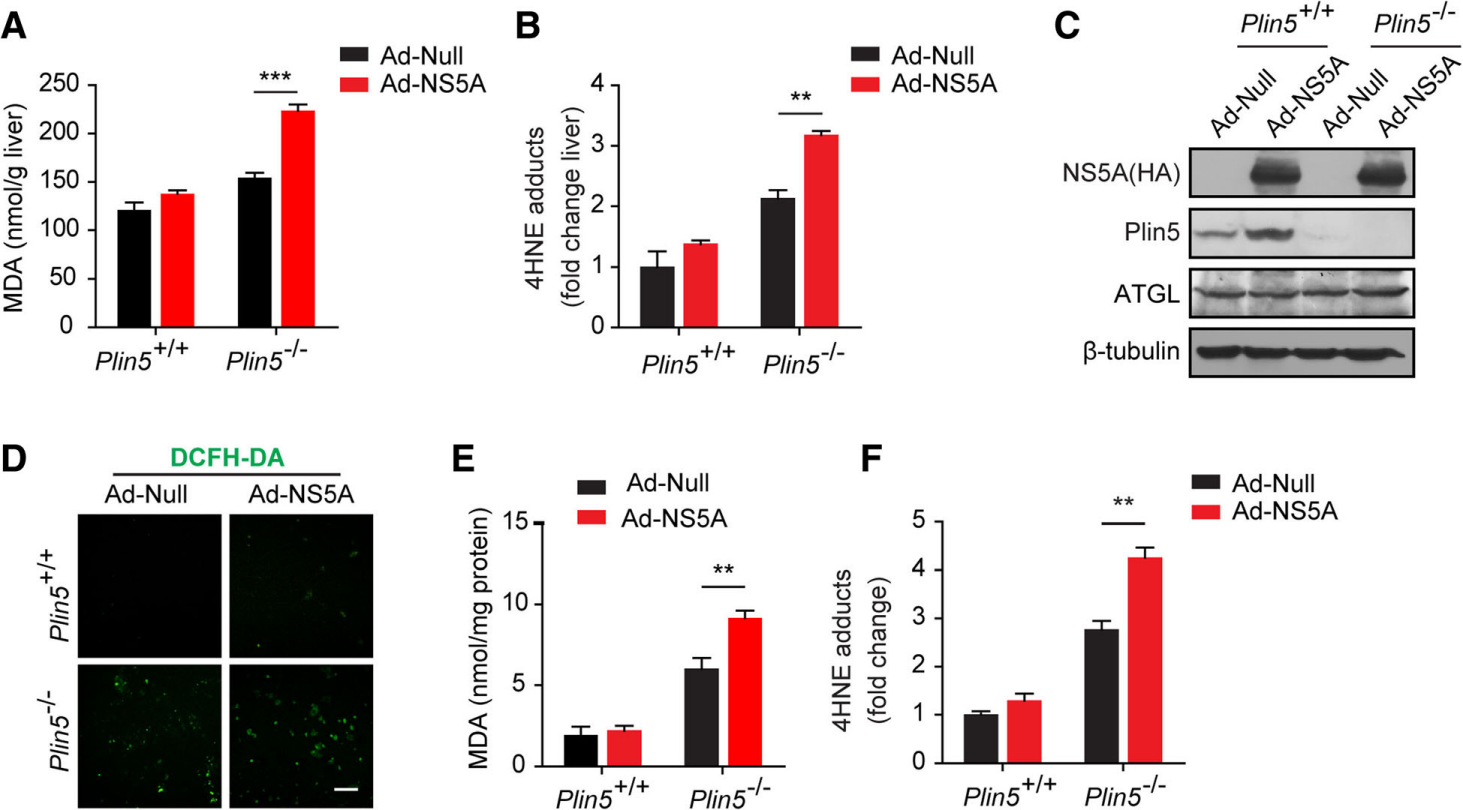
Plin5 alleviated NS5A-induced lipid peroxidation in liver cells. **a** and **b** Livers were isolated and homogenized, and the levels of MDA and 4-hydroxynonenal (4-HNE), two lipid oxidation products, were determined in the homogenate (*n* = 4). **c** Primary hepatocytes were isolated from wild-type (*Plin5*^+/+^) and *Plin5*-null (*Plin5*^−/−^) mice and infected with control (Ad-Null) and NS5A-expressing (Ad-NS5A) adenoviruses. The expression of NS5A, Plin5 and ATGL in primary hepatocytes was analysed by immunoblotting. **d** After treatment with 200 μM oleic acid (OA) for 24 h, the ROS levels in hepatocytes with or without NS5A expression were determined by DCFH-DA staining. Scale bar = 50 μm. **e** and **f** The levels of MDA and 4-hydroxynonenal (4-HNE) were analysed in primary hepatocytes with or without NS5A expression.**P < 0.01, ***P < 0.001. Data are means ± SEs for triplicate samples for each representative experiment

Enhanced ROS production is common in liver injuries with HCV infection, and can induce the lipid peroxidation process. The most relevant biomarkers are malondialdehyde (MDA) and 4-hydroxynonenal (4-HNE) [20, 21]. Here, NS5A expression was responsible for increased MDA and 4-HNE levels in the *Plin5*-null liver, and slightly increased 4-HNE levels were observed in wide-type mice (Fig. 2a &b). Primary hepatocytes were isolated from the livers of wild-type and *Plin5*-null mice and infected with Ad-NS5A and Ad-Null. Immunoblotting verified that NS5A was expressed in both wild-type and *Plin5*-null hepatocytes, and the expression of ATGL remained unaltered (Fig. 2c). The ROS levels in primary hepatocytes were evaluated by DCFH-DA staining. As shown in Fig. 2d, NS5A significantly induced ROS production in *Plin5*-null primary hepatocytes but slightly altered ROS levels in the wild-type hepatocytes compared to the ROS levels in Ad-Null-treated mice (Fig. 2d). Moreover, NS5A tremendously elevated the levels of MDA and 4-HNE in the *Plin5*-null hepatocytes compared to those in wild-type hepatocytes, indicating accelerated lipid peroxidation. However there was no indication of a steep increase in lipid peroxidation in NS5A-expressing wild-type hepatocytes compared with that in control group (Fig. 2e &f). These results suggested that Plin5 alleviated NS5A-induced lipid peroxidation in the liver.

### Plin5 promoted lipid accumulation and reduced the lipolysis induced by NS5A

**Fig. 3:**
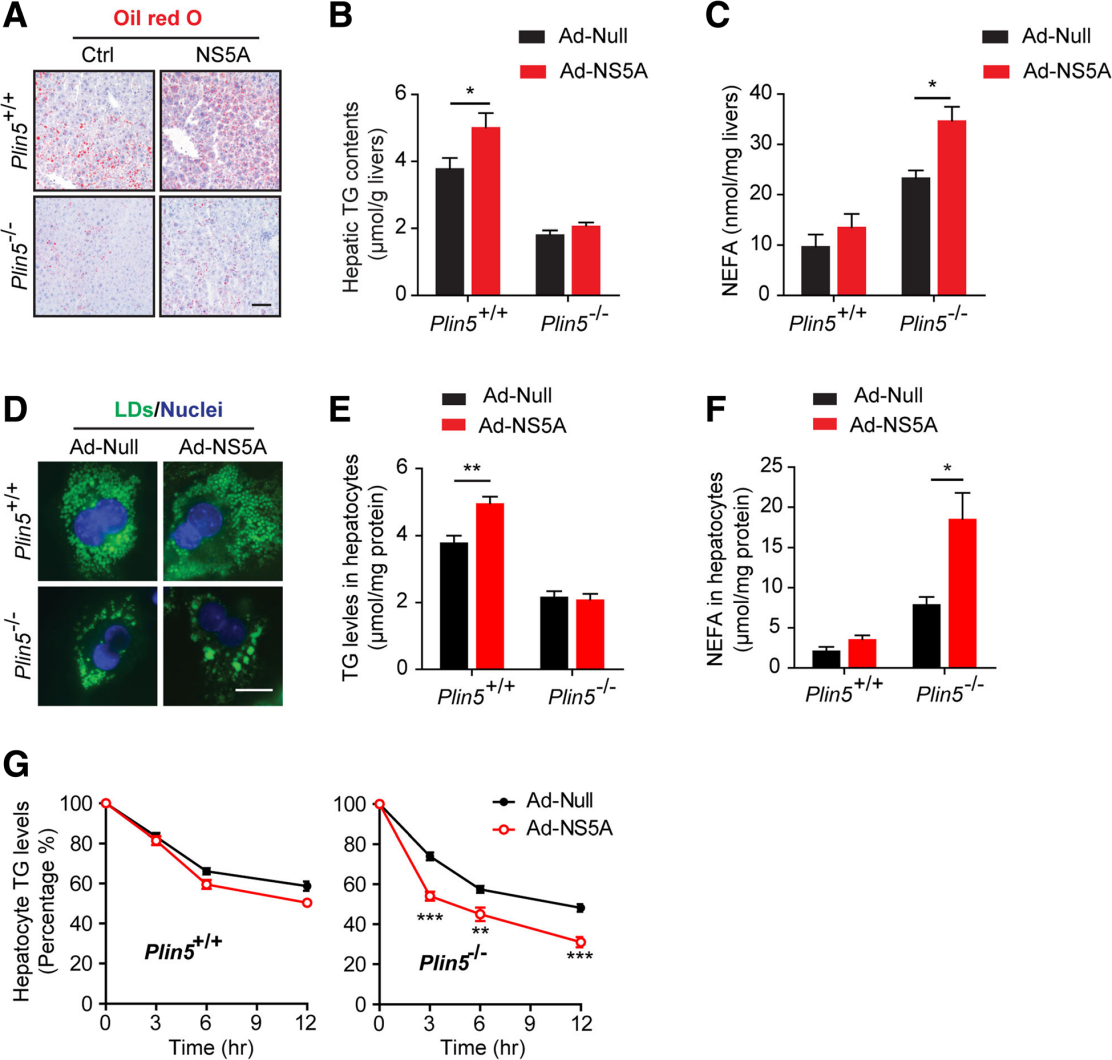
Plin5 stimulated lipid accumulation and reduced the lipolysis induced by NS5A. **a** Oil red O (ORO) staining of liver tissues from wild-type (Plin5^+/+^) and *Plin5*-null (Plin5^−/−^) mice. Scale bar = 50 μm. **b** & **c** Triglyceride (TG) and non-esterified fatty acid (NEFA) content was quantified by commercial kits after extraction with hexane/isopropanol. **d** Primary hepatocytes were isolated from wild-type and *Plin5*-null mice and then infected with control (Ad-Null) and NS5A-expressing (Ad-NS5A) adenoviruses. The lipid droplets (LDs) in primary hepatocytes were stained with BODIPY 493/503 and observed under a fluorescence microscope. Scale bar = 20 μm. **e** After extraction with hexane/isopropanol, the TG content was quantified in wild-type (Plin5^+/+^) and *Plin5*-null (Plin5^−/−^) hepatocytes. **f** Primary hepatocytes with or without NS5A expression were treated with triacsin C (5 μM), an inhibitor of acyl-CoA synthase, and the NEFA content was determined over 8 h. **g** Wild-type (*Plin5*^+/+^) and *Plin5*-null (*Plin5*^−/−^) primary hepatocytes with or without NS5A expression were incubated with 200 μM OA for 24 h. The cells were then treated with 5 μM triacsin C to inhibit further triacylglycerol synthesis. Cellular lipids were extracted with hexane/isopropanol, and then the TG content was quantified. *P < 0.05, **P <0.01, ***P < 0.001. Data are means ± SEs for triplicate samples for each representative experiment

Similar to core antigen, NS5A was reported to increase the deposition of neutral lipids and maintain a lipid-rich environment. After tail vein injection with NS5A adenovirus, the oil red O staining illustrated that NS5A increased the lipid content in the presence of Plin5 but not in the *Plin5*-null livers (Fig. 3a). The results of quantitative determination of the triglyceride (TG) content coincided with those of ORO staining, which indicated that Plin5 played a crucial role in the NS5A-induced lipid accumulation (Fig. 3b). Simultaneously, we also found that the non-esterified fatty acid (NEFA) levels were higher in livers expressing NS5A than those that did not express NS5A, and this elevation was aggravated in the livers of *Plin5*-null mice (Fig. 3c). In vitro, BODIPY 493/503 staining suggested that NS5A also fuelled TG deposition in wild-type hepatocytes and that Plin5 deficiency significantly impeded lipid accumulation in NS5A-expressing hepatocytes (Fig. 3d). Consistent with this, TG quantification showed a similar tendency (Fig. 3e). Moreover, Plin5 deficiency exacerbated the elevation of NEFAs in primary hepatocytes (Fig. 3f). Abundant in liver cells, Plin5 protected the LDs against lipolysis. As an inhibitor of acyl-CoA synthase (an enzyme critical to fatty acid synthesis), triacsin C blocks TG biosynthesis. The relative lipid lipolysis rate can be measured by detecting the residual TG content after triacsin C treatment. Here, we found that NS5A only slightly reduced the TG content in wild-type hepatocytes, but significantly reduced the TG content in *Plin5*-null hepatocytes in the presence of Triacsin C (Fig. 3g), which indicated that Plin5 deficiency aggravated the NS5A-induced lipolysis of LDs. These results indicated that NS5A induced lipid dysmetabolism and Plin5 reduced NS5A-induced lipolysis in the liver.

### Plin5 overexpression blunted peroxidation injury induced by NS5A

**Fig. 4:**
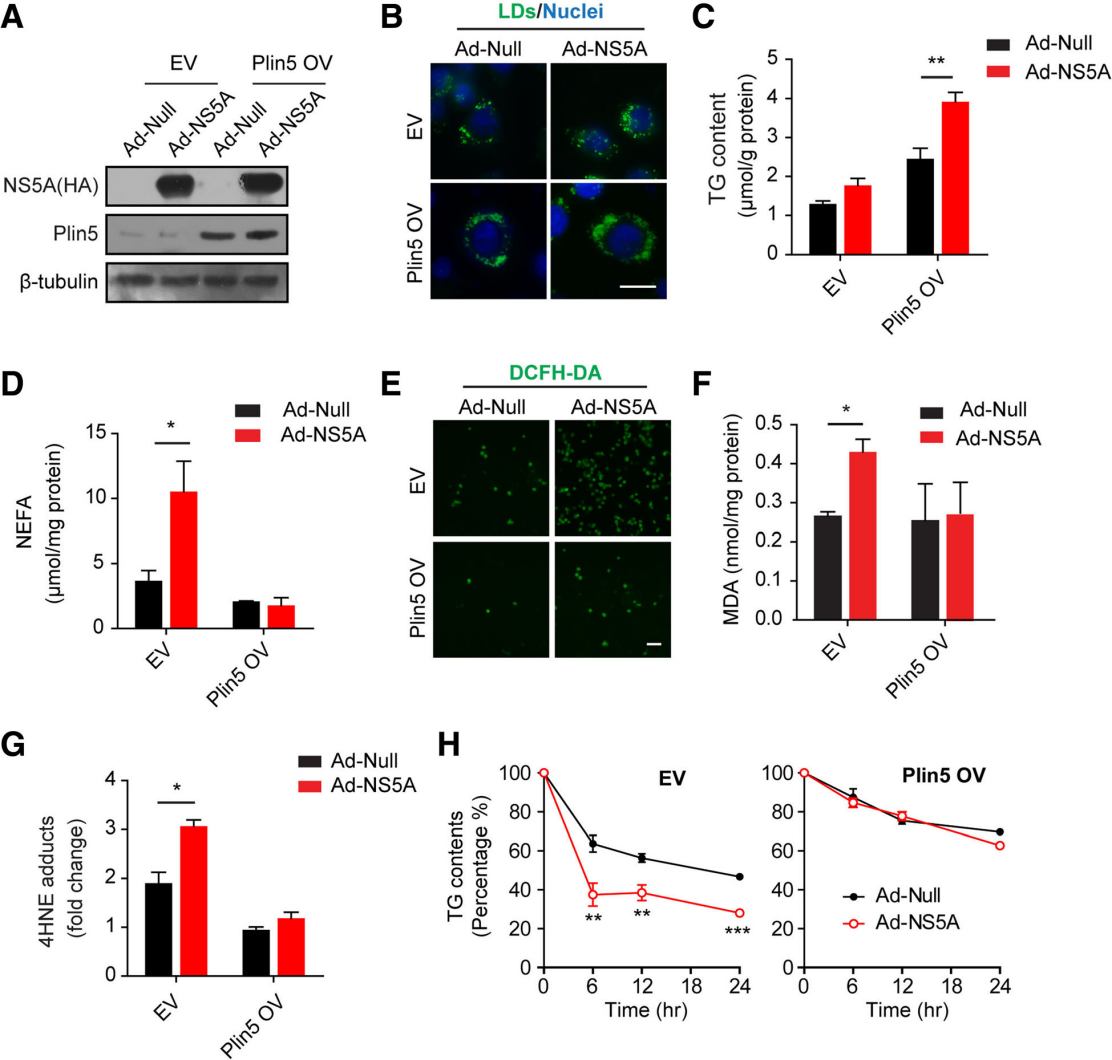
Plin5 overexpression blunted peroxidation injury induced by NS5A. **a** HepG2 cells expressing Plin5 (Plin5 OV) and empty vehicle (EV) were established by lentivirus infection. The expression of NS5A and Plin5 in HepG2 cells was analysed by immunoblotting. (**b** & **c**) The influence of NS5A on lipid content was observed in HepG2 cells with or without Plin5 overexpression after BODIPY 493/503 staining (**b**) and TG quantification (**c**). Scale bar = 10 μm. **d** Intracellular NEFAs were quantified by commercial kit after extraction with hexane/isopropanol. **e** HepG2 cells with or without Plin5 overexpression were infected with control (Ad-Null) and NS5A-expressing (Ad-NS5A) adenoviruses. After treatment with 200 μM oleic acid (OA) for 24 h, the ROS levels in HepG2 cells were observed by DCFH-DA staining. Scale bar = 50 μm. **f** & **g** The levels of MDA and 4-hydroxynonenal (4-HNE), two lipid oxidation products, were analysed in HepG2 cells with or without NS5A expression. **h** The triacylglycerol hydrolysis rate in HepG2 cells with or without NS5A expression was determined in the presence of triacsin C (5 μM). *P < 0.05, **P <0.01, ***P < 0.001. Data are means ± SEs for triplicate samples for each representative experiment

To confirm the results obtained in the liver and primary hepatocytes, NS5A was expressed in HepG2 cells with or without Plin5 overexpression. Immunoblotting results verified the overexpression of Plin5 and NS5A in HepG2 cells (Fig. 4a). BODIPY 493/503 staining showed that the NS5A protein increased the lipid content in HepG2 cells with or without Plin5 overexpression (Fig. 4b). TG quantification also indicated that Plin5 overexpression greatly increased the lipid accumulation induced by NS5A in HepG2 cells (Fig. 4c). In parallel with lipid deposition, NS5A increased NEFA levels in HepG2 cells, but the overexpression of Plin5 diminished the NEFA elevation in HepG2 cells (Fig. 4d). Similarly to the results in primary hepatocytes, the DCFH-DA staining showed that NS5A consistently elevated ROS levels, and that Plin5 overexpression blunted the ROS elevation induced by NS5A in HepG2 cells (Fig. 4e). Consistent with this, Plin5 overexpression blunted the elevation of MDA and 4-HNE levels induced by NS5A in HepG2 cells (Fig. 4f &g). Therefore, we also analysed the TG hydrolysis rate in the presence of triacsin C. The results indicated that NS5A elevated TG lipolysis in HepG2 cells transformed with the empty vector (EV), however, Plin5 overexpression (Plin5 OV) inhibited the NS5A-induced increase in lipolysis in HepG2 cells (Fig. 4h). Thus, Plin5 overexpression in HepG2 cells ameliorated NS5A-induced lipolysis and lipotoxic injuries.

### NS5A recruited Plin5 to lipid droplets by direct interaction

**Fig. 5:**
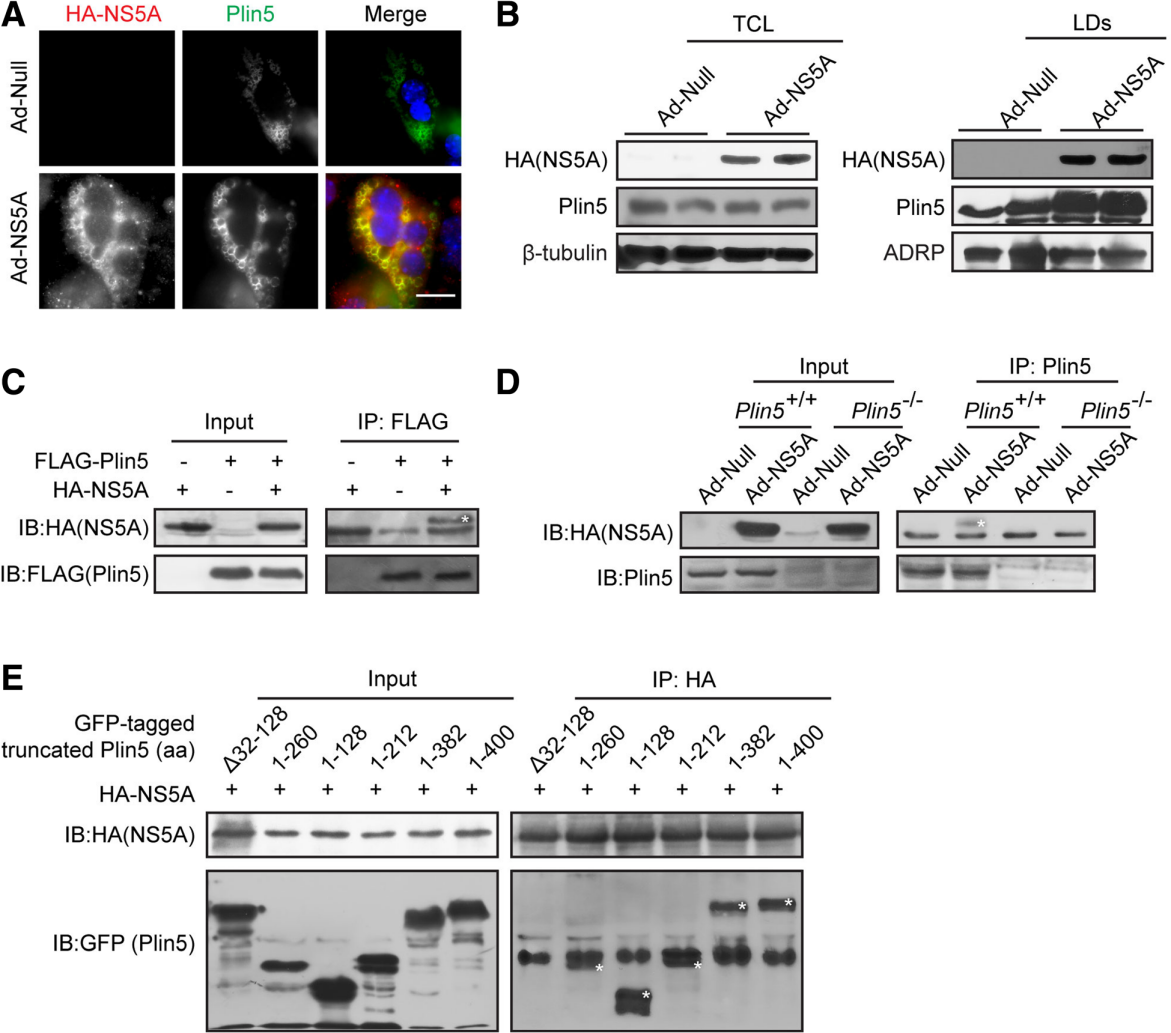
NS5A recruited Plin5 to LDs. **a** NS5A was expressed in primary hepatocytes, and exogenous HA-NS5A (red) and endogenous Plin5 (green) were observed by immunofluorescent staining. Scale bar = 20 μm. **b** LD-containing fractions were isolated from wild-type mice with or without NS5A expression by using sucrose density gradient ultracentrifugation, and the Plin5 content was determined in both the total cell lysate (TCL) and the lipid droplet (LD)-containing fraction by immunoblotting. **c** Co-IP assay was performed to determine the interaction of exogenous HA-tagged NS5A and FLAG-tagged Plin5 in 293 T cells. Cell lysates were subjected to immunoprecipitation with anti-FLAG antibody and then immunoblotted with an anti-HA antibody. **d** NS5A was expressed in wild-type (*Plin5*^+/+^) and *Plin5*-null (*Plin5*^−/−^) primary hepatocytes, endogenous Plin5 was immunoprecipitated with anti-Plin5 antibody, and NS5A was detected by immunoblotting with anti-HA antibody. **e** Co-IP assays to identify the specific domain of Plin5 responsible for the Plin5-NS5A interaction. Truncated GFP-tagged Plin5 constructs were overexpressed in 293 T cells with HA-tagged NS5A. NS5A was immunoprecipitated by anti-HA monoclonal antibody, and truncated Plin5 was detected by immunoblotting

To further investigate the role Plin5 plays in NS5A-induced hepatic injury, the subcellular localization of Plin5 in NS5A-expressing primary hepatocytes were analysed. Immunofluorescent staining demonstrated that NS5A co-localized with Plin5 on the LDs (Fig. 5a). However, we did not observe a remarkable change in the protein levels of Plin5 in whole cell lysates, which indicated NS5A did not affect the expression of Plin5. Furthermore, immunoblotting revealed that the protein levels of Plin5 were significantly elevated in the LD-containing fractions of NS5A-expressing hepatocytes, which suggested that NS5A expression recruited Plin5 to LD-containing fractions (Fig. 5b). Moreover, the direct interaction between NS5A and Plin5 was analysed by co-immunoprecipitation assay. HEK 293 T cells were used for co-transfection of the plasmids expressing FLAG-tagged Plin5 and HA-tagged NS5A, and NS5A was pulled down by anti-FLAG M2 affinity gel. Immunoblotting showed that NS5A was co-immunoprecipitated with Plin5 (Fig. 5c). Furthermore, NS5A was expressed in the wild-type and *Plin5*-null primary hepatocytes respectively. Plin5 was immunoprecipitated and the immunoblotting results showed that the NS5A protein co-immunoprecipitated with endogenous Plin5 (Fig. 5d). To determine which domain of Plin5 interacts with NS5A, GFP-tagged Plin5 truncations were overexpressed in 293 T cells expressing NS5A, we found that all of the truncated Plin5 constructs containing the N-terminal 1–128 amino acids (aa) interacted with NS5A except for a truncated 32–128 aa deletion construct (Fig. 5e), which indicated that the N-terminal 32–128 aa of Plin5 are pivotal for its binding with NS5A. Thus, these results suggested that NS5A interacted with Plin5, and then recruited cytoplasmic Plin5 to LDs.

## Discussion

As is known to all, lipid droplets (LDs) are ubiquitous organelles in most eukaryotic cells and in some prokaryotes. Serving as lipid reservoirs, LDs plays important roles in storing neutral lipids, mainly triglycerides (TGs) and cholesterol esters (CEs) [22, 23]. Emerging evidence has revealed that LDs are integral to lipid metabolism, contributing to both lipid synthesis and consumption. Recently, LDs have attracted increasing interest because of their important role in disease progression.

Involved in HCV infection, LDs occupy a central position in producing infectious viruses through their interactions with various host and viral proteins. As previously reported, HCV RNA is replicated in “membranous web”, and the replication complex is then recruited to adjacent LDs in a core-dependent way [5]. In proximity to LDs, the viral RNA is intimately associated with the HCV non-structural proteins, host proteins and ER membranes [24–26] to form infectious virions. Among all non-structural proteins involved in the process of HCV replication, NS5A plays a critical role. The most important feature of chronic HCV infection is disordered lipid metabolism. HCV viruses manipulate host lipid metabolism for their own life cycle and predispose chronic HCV patients to steatosis. HCV infection or the core protein alone induces the expression of sterol regulatory element binding protein-1c (SREBP-1c) [27] and its downstream targets including fatty acid synthase (FASN) [28]. In addition, NS5A increases the intracellular triacylglycerol content by transcriptionally elevating PPAR-γ [8], thus accelerating lipid biosynthesis, but its role in hepatic lipid dysmetabolism still needs further investigation.

To analyse how NS5A regulates the intracellular lipid content, we found that NS5A stimulated TG accumulation in the presence of Plin5 but significantly elevated NEFA levels in *Plin5*-deficient liver cells. A previous study demonstrated that Plin5 alone facilitated lipid storage [15]. As a member of the perilipin family, Plin5 is abundant in the liver and is elevated under lipid overload conditions, thus preventing peroxidation injury induced by excessive lipolysis via interaction with adipose triglyceride lipase (ATGL), comparative gene identification-58 (CGI-58) [29] and hormone-sensitive lipase (HSL) [30]. Plin5 is considered to be an energy sensor that regulates the flux of fatty acids (FAs) to meet cellular energetic demands and to protect mitochondria from excess peroxidation. Based on the elevated lipolysis rate induced by NS5A, we also analysed the expression of ATGL, but no distinguishable changes were detected in both wild-type and *Plin5*-null livers. Collectively, the results of our study suggested that NS5A maintains a lipid-rich environment with the assistance of Plin5.

Excess lipolysis often leads to elevated lipid peroxidation. In this study, HCV NS5A expression significantly increased excessive lipid peroxidation and inflammation in the liver, which were aggravated in the *Plin5*-null mice. Also, the serum levels of TNF-α and IL-2 were significantly elevated in the NS5A-expressing *Plin5*-null mice. Lipid peroxidation-derived aldehydes (LDAs) are a series of inflammatory pathological products that accompany with various ROS. Among them, the most common LDAs are malondialdehyde (MDA) and 4-hydroxynonenal (4-HNE). In addition, NS5A associated with the endoplasmic reticulum (ER), and the mechanism underlying this association was attributed to subsequently enhanced mitochondrial ROS production [31]. Previous evidence also suggested that the NF-κB/AP-1 pathway has an intimate link with viral proteins including the core protein as well as the non-structural proteins NS3 and NS5A [32]. However, in vitro Plin5 overexpression reduced the level of MDA and 4-HNE induced by NS5A. Thus, Plin5 protected the liver from peroxidation and the subsequent inflammatory injuries induced by NS5A.

HCV NS5A disturbed hepatic lipid metabolism by interacting with various lipid droplet proteins such as Cideb [33] and DGAT1 [34], which contributed to increased lipid deposition in cells. Here, we found that NS5A also directly interacted with Plin5. As a vital member of the perilipin family, Plin5 is anchored on the surface of LD and shares high sequence homology of its N terminus with other family members. Here, co-immunoprecipitation revealed that Plin5 interacted with NS5A through its PAT-1 domain. In Plin5, the PAT-1 domain was indispensable for LD binding [35], facilitating the targeting of cytoplasmic Plin5 to LDs. Thus, we concluded that HCV NS5A promoted Plin5 localization on LDs, and the underlying mechanism may be attributed to the NS5A-Plin5 interaction, which prompts the transfer of Plin5 from the cytoplasm to the surface of LDs. This explains why Plin5 deficiency aggravated hepatic lipotoxic injuries in NS5A-expressing livers.

Plin5 not only localizes to the cytosol and LDs but also interacts with mitochondria [36, 37], which implies its divergent roles in lipid metabolism. A cluster of studies in immortalized and primary cells including hepatocytes, skeletal muscle and cardiomyocytes revealed that Plin5 served as a protective factor against lipotoxic injury through increasing FA uptake [38], decreasing lipolysis [13] and promoting FA oxidation [38]. Our observations favour the interpretation that Plin5 recruited to LDs protects against NS5A-induced catabolism syndrome.

## Conclusions

In this study, we provide evidence that NS5A recruited cytoplasmic Plin5 to the surface of LDs, facilitating its metabolic need for a lipid-rich environment. Simultaneously, our data indicated the protective role of Plin5 against hepatic lipotoxic injuries induced by HCV NS5A, which is helpful to understand steatosis and injuries of the liver with HCV infection.

## Abbreviations

4-HNE: 4-hydroxynonenal
ATGL: Adipose triglyceride lipase
CGI-58: Comparative gene identification-58
CideB: Cell death-inducing DFFA-like effector B
DGAT1: Diglyceride acyltransferase 1
FA: Fatty acid
FASN: Fatty acid synthase
HCV: Hepatitis C virus
HSL: Hormone-sensitive lipase
LDs: Lipid droplets
MDA: Malondialdehyde
NEFA: Non-esterified fatty acid
NS5A: Non-structural protein 5A
Plin5: Perilipin 5
ROS: Reactive oxygen species
SREBP-1c: Sterol regulatory element binding protein-1c
TIP47: Tail-interacting protein 47
